# Alginate-Based Composite Sponges as Gastroretentive Carriers for Curcumin-Loaded Self-Microemulsifying Drug Delivery Systems

**DOI:** 10.3390/scipharm85010011

**Published:** 2017-03-15

**Authors:** Arpa Petchsomrit, Namfa Sermkaew, Ruedeekorn Wiwattanapatapee

**Affiliations:** 1 Department of Pharmaceutical Technology, Faculty of Pharmaceutical Sciences, Prince of Songkla University, Hat-Yai, Songkhla 90112, Thailand; Arpa@buu.ac.th; 2 Phytomedicine and Pharmaceutical Biotechnology Excellence Research Center, Faculty of Pharmaceutical Sciences, Prince of Songkla University, Hat-Yai, Songkhla 90112, Thailand; 3 Drug and Cosmetic Research and Development Unit, School of Pharmacy, Walailak University, Nakhon Si Thammarat 80161, Thailand; namfa.se@wu.ac.th

**Keywords:** curcumin, self-microemulsifying drug delivery systems, sponge, alginate

## Abstract

Alginate-based composite sponges were developed as carriers to prolong the gastric retention time and controlled release of curcumin-loaded self-microemulsifying drug delivery systems (Cur-SMEDDS). Liquid Cur-SMEDDS was incorporated into a solution made up of a mixture of polymers and converted into a solid form by freeze-drying. The ratio of alginate as the main polymer, adsorbent (colloidal silicon dioxide), and additional polymers—sodium carboxymethyl cellulose (SCMC), hydroxypropyl methylcellulose (HPMC)—was varied systematically to adjust the drug loading and entrapment efficiency, sponge buoyancy, and the release profile of Cur-SMEDDS. The optimum composite sponge was fabricated from a 4% alginate and 2% HPMC mixed solution. It immediately floated on simulated gastric fluid (SGF, pH 1.2) and remained buoyant over an 8 h period. The formulation exhibited an emulsion droplet size of approximately 30 nm and provided sustained release of Cur-SMEDDS in SGF, reaching 71% within 8 h compared with only 10% release from curcumin powder. This study demonstrates the potential of alginate-based composite sponges combined with self-microemulsifying formulations for gastroretention applications involving poorly soluble compounds.

## 1. Introduction

Curcumin, a natural polyphenol derived from the rhizome of turmeric (*Curcuma longa* Linn.), has been widely used in traditional medicine for centuries. Although curcumin exerts various biological effects, such as antioxidative [1], anti-inflammatory [2,3], antimicrobial [4], antiproliferative [5], and anticancer effects [6], the use of curcumin is limited by its poor aqueous solubility (30 pmol/mL) [7] and rapid metabolism, which result in low oral bioavailability [8].

Numerous drug delivery system designs have been investigated with the aim of improving the oral bioavailability of curcumin, including nanoparticles [9] and liposomes [10]. Self-microemulsifying drug delivery systems (SMEDDS) have been studied extensively for enhancing the oral bioavailability of highly lipophilic compounds, such as curcumin and tetrahydrocurcumin [11,12,13,14]. Self-microemulsifying drug delivery systems are typically produced in liquid form but are recognized as having several limitations, such as leakage from capsules and drug precipitation during storage, which reduces the effective dose contained within the microemulsion [15]. Solid SMEDDS (S-SMEDDS) have been developed to overcome the limitations of liquid formulations by combining SMEDDS with various pharmaceutical excipients to create solid dosage forms, such as tablets [12], pellets [11], powders [16], granules [17], beads [13], and microcapsules [18].

Prolongation of gastric retention time, coupled with controlled release of curcumin, is advantageous during treatment for gastrointestinal diseases including peptic ulcers or gastrointestinal cancer [19]. To elevate the solubility of curcumin, SMEDDS has been one of the most successful methods [11,13,14]. The gastric residence time of curcumin can be also prolonged by using suitable gastroretentive drug delivery systems. The gastroretentive drug delivery systems are designed to exhibit a lower density than the gastric contents (1.004–1.010 g/cm^3^) and to remain floating in the stomach for prolonged time periods without affecting the rate of gastric emptying [20,21]. In addition, the dosage form is expected to provide sustained and efficient drug release during residence in the stomach. 

Microporous polymeric materials, or sponges, are attractive for gastroretentive drug delivery applications due to their inherent low-density and flotation properties, and their porous structure, which offers opportunities for achieving efficient drug release. In addition, sponge manufacture generally involves relatively few components and simple procedures compared with other previous floating solid dosage forms such as beads and tablets, for which effervescent materials are needed to provide their floating properties [12,13,22]. The incorporation of curcumin-loaded SMEDDS within a floating sponge offers a novel formulation for local treatment of gastrointestinal diseases. Hydroxypropyl methylcellulose (HPMC)-based sponges have recently been investigated as a carrier for oral delivery of self-microemulsifying curcumin [14]. Although in vivo oral absorption of curcumin in rabbit (2615.68 ng·h/mL) was significantly enhanced by these systems compared with free curcumin (509.49 ng·h/mL), immediate release of curcumin occurred, which is anticipated to result in high dosing frequencies and, subsequently, reduced patient compliance in practice. 

Alginate is a naturally occurring polysaccharide derived from seaweed and comprises mannuronic acid (M) and guluronic acid (G) monomers. These monomers can be rearranged in homopolymeric blocks of consecutive G-residues (G-blocks), M-residues (M-blocks), or heteropolymeric blocks (MG-blocks). The molecular compositions of alginates are generally referred to as high M or high G. The block sections exhibit different chain flexibilities in solution. Most commercial grades are of the high-M type, which provide more elastic gels. The excellent biocompatibility, biodegradability, and broad regulatory acceptance of alginate has resulted in their use in a wide variety of pharmaceutical applications as drug delivery vehicles in the form of microcapsules, pellets, and beads to modify drug release profiles during oral controlled drug delivery. Sodium alginate, under acidic conditions, is converted to a water-insoluble, compact alginic acid hydrogel that controls the release of incorporated drugs, including tetrahydrocurcumin and β-lapachone [11,23]. The objective of this study was to investigate the potential of a new drug delivery system, floating alginate-based sponges, as a gastroretentive device for SMEDDS formulation of curcumin.

## 2. Materials and Methods

### 2.1. Materials

Alginate (high-M type, low viscosity) and curcumin were purchased from Sigma–Aldrich (Buchs, Switzerland). Capryol 90 (propylene glycol monocaprylate), Labrafac PG (propylene glycol caprylate/caprate), and Labrasol (caprylocaproyl macrogol-8 glycerides) were obtained from Gattefossé (Saint-Priest, France). Cremophor EL (polyoxyethylene castor-oil derivatives) was purchased from BASF (Ludwigshafen, Germany). Aerosil 200 (colloidal silicon dioxide) was obtained from Degussa-Hüls AG (Hanau, Germany). Sodium carboxymethyl cellulose (SCMC, high viscosity) was obtained from PC Drug Center Co., Ltd. (Bangkok, Thailand). Hydroxypropyl methylcellulose–HPMC A15 LV; 29.5% methoxy and viscosity of 15 mPa·s (2% in water at 20 °C) and HPMC A4C; 29.5% methoxy and viscosity of 400 mPa·s (2% in water at 20 °C))–was provided by Colorcon (Indianapolis, IN, USA). Hard gelatin capsules (size 00) were supplied by Capsugel (Bangkok, Thailand). Methanol was obtained from RCI Labscan (Bangkok, Thailand). All other chemicals were of analytical grade.

### 2.2. Preparation of Liquid Curcumin-Loaded Self-Microemulsifying Drug Delivery Systems 

Liquid curcumin-loaded self-microemulsifying drug delivery systems (Cur-SMEDDS) was prepared in accordance with our previous study [11]. In brief, a mixture of liquid components –Cremophor EL (315 mg), Labrasol (315 mg), Capryol 90 (135 mg), and Labrafac PG (135 mg)– was stirred until homogeneous. Curcumin powder (40 g) was dispersed in the liquid mixture using a magnetic stirrer until a uniform solution was obtained. The Cur-SMEDDS formulation was stored in tightly sealed glass bottles at room temperature before use.

### 2.3. Preparation of Alginate-Based Sponges Loaded with Curcumin-Loaded Self-Microemulsifying Drug Delivery Systems

#### 2.3.1. Alginate Sponges

Separate alginate solutions with specific concentrations in the range of 3%–5% *w*/*w* were prepared in distilled water. A weighed amount of Cur-SMEDDS was added to the solution with continuous stirring to form an oil in water (*o*/*w*) emulsion with Cur-SMEDDS loading of 5%–20% *w*/*w*. The emulsion was poured into 96-well plates and air bubbles were removed by storing at 5 °C overnight. The resulting solidified material was freeze-dried (Martin Christ, Osterode am Harz, Germany) and stored in a desiccator prior to use.

#### 2.3.2. Alginate-Colloidal Silicon Dioxide Sponges

Colloidal silicon dioxide (used as an adsorbent) was dispersed into 4% *w*/*w* alginate solution with constant agitation using a magnetic stirrer. The *w*/*w* ratio of Cur-SMEDDS to colloidal silicon dioxide was maintained at 5:1 and 10:1. Then, Cur-SMEDDS was added to the dispersion of colloidal silicon dioxide in alginate to obtain a specific loading between 15% and 25% *w*/*w* in a total preparation weight of 100 g. The mixture was stirred until homogeneous, and Cur-SMEDDS loaded alginate-colloidal silicon dioxide sponges were produced as described above.

#### 2.3.3. Alginate-Based Composite Sponges

Individual solutions of SCMC and HPMC with specific concentration between 1% and 3% *w*/*w* concentration were prepared in distilled water. Alginate solution (4% *w*/*w*) was blended with SCMC or HPMC solution (1%–3% *w*/*w*) and Cur-SMEDDS was added to the polymer blend to obtain a loading of 15% *w*/*w* in a 100 g mixture. The mixtures were stirred continuously using a magnetic stirrer to produce an *o*/*w* emulsion and alginate-based composite sponges were prepared by storage at 5 °C followed by lyophilization as described above.

### 2.4. Investigation of Morphology

The surface internal structure of the alginate-based sponges was examined using scanning electron microscopy (SEM). Scanning electron microscopy micrographs (six images per sponge and 100 different pores) were analyzed to determine mean pore size [24,25]. The morphology of the SMEDDS within the alginate-based sponges was assessed using transmission electron microscopy (TEM) (JEOL Ltd., Tokyo, Japan). Samples of sponges (containing the equivalent of 1 g of liquid Cur-SMEDDS) were introduced into 75 mL of simulated gastric fluid (SGF)–0.1 N hydrochloric acid (HCl),75-fold dilution—at room temperature, and gently stirred for 8 h. The content of the SGF was then filtered and placed on copper grids. Filter paper was used to absorb the excess fluid and samples were coated with 2% phosphotungstic acid for 10 min. The overload fluid was removed and the sample grids were examined by TEM after drying [12,26,27].

### 2.5. Weight Variation

Twenty sponges from each formulation were selected at random and weighed individually using a digital balance (Denver Instrument, Bohemia, NY, USA) to calculate the average sample weight. The formulation was considered to be consistent if no more than two of the individual weights deviated from the average weight by more than 5% [28].

### 2.6. Emulsion Droplet Size Analysis

Alginate-based sponges, containing the equivalent of 4 mg curcumin, were placed in 20 mL of SGF (5-fold dilution) at room temperature, and gently stirred for 8 h. The content was filtered through a 0.45 μm membrane filter and the droplet size and polydispersity index (PDI) of the microemulsion were verified using a Zetasizer Nano ZS, Zeta potential and particle size analyzer (Malvern Instruments, Malvern, UK). Dynamic light scattering measurements were obtained at a fixed angle of 90° and a temperature of 25 °C [12]. Triplicate samples were each analyzed for 1 min, and for each measurement ran, 12 sub-runs were performed. The data are presented as the mean ± standard deviation (SD).

### 2.7. Drug Content and Drug Entrapment Efficiency

Samples of alginate-based sponges (0.1 g) were weighed accurately, introduced into 50 mL of methanol, and sonicated for 90 min. The solution was filtered through a 0.45 µm membrane filter and analyzed for curcumin content using UV–vis spectrophotometry (Thermo Spectronic genesys 5, Haverhill, MA, USA) at 425 nm. The actual amount of curcumin was calculated from the absorbance value, by comparison with the linear equation derived using a serial dilution of curcumin in methanol [28,29]. Each formulation was analyzed in triplicate. The curcumin entrapment efficiency (EE) of sponges loaded with Cur-SMEDDS was also calculated with respect to the initial content of curcumin in the sponges [29,30] according to Equation (1):

(1) EE =A/B × 100%

where *A* is the measured amount of curcumin in the sponges and *B* is the initial amount contained in the formulation. Data are presented as the mean ± SD (*n* = 3).

### 2.8. Water Sorption Capacity of Alginate-Based Sponges

The water adsorption capacities of alginate-based sponges were evaluated by immersing samples in 10 mL of SGF at 37 ± 0.5 °C. Samples were removed from the medium at time intervals of 30, 60, 90, 120, 180, 240, 300, 360, 420, and 480 min, and excess fluid was carefully removed using filter paper. The samples were weighed immediately using a digital balance [21] and the percentage water adsorption of the sponge (*W_ad_*) was calculated using Equation (2):

(2) Wad = (Wt − Wo)/Wo × 100

where *Wt* represents the sample weight at a particular immersion time in SGF and *Wo* is the initial weight. Values are expressed as the mean ± SD (*n* = 20).

### 2.9. Buoyancy

Samples of alginate-based sponge were added to 70 mL SGF that was maintained at 37 °C and gently stirred at 75 rpm using a magnetic stirrer. The lag time to float and the floating behavior of the sponges in the test medium were recorded for a period of 8 h. The time taken for the samples to float to the top of the medium was defined as floating lag time, and the time period over which the sponges floated on the surface of the medium was classified as floating duration [27,31].

### 2.10. In Vitro Drug Release from Alginate-Based Sponges

In vitro drug release of curcumin from alginate-based sponges was monitored using a USP XXIII Dissolution Apparatus II (paddle type) (Hanson Research Corporation, Chatsworth CA, USA). Hard gelatin capsules filled with sponges (0.1 g) were added to 450 mL of SGF at 37 ± 0.5 °C, and the rotation speed was set at 50 rpm [31,32]. Aliquots of release medium (5 mL) were withdrawn at 30, 60, 90, 120, 180, 240, 300, 360, 420, and 480 min, and replaced by an equal volume of fresh medium. The samples were filtered using a 0.45 μm filter, and the amount of drug released was quantified spectrophotometrically at a wavelength of 425 nm by comparison with a standard curve of absorbance versus curcumin concentration. Each formulation was tested in triplicate, and data were presented as the mean ± SD. A plot of percentage cumulative release of curcumin against time was constructed to demonstrate the release profile.

### 2.11. Stability Studies

Stability testing of alginate-based sponge samples, prepared under optimized formulation conditions, was carried out for 6 months according to the International Conference on Harmonisation (ICH) guidelines Q 1 A (R2), which are concerned with stability testing of new drug substances and products. “Intermediate” testing conditions were applied (30 ± 2 °C/65% ± 5% relative humidity (RH)), as well as “accelerated” conditions (45 ± 2 °C/75% ± 5% RH) [33]. Capsules filled with sponges were placed in a tightly sealed glass container and stored within a constant climate chamber (Memmert HPP 260, Schwabach, Germany) with humidity and temperature controlled under both conditions. After 3 and 6 months, the optimized sponges loaded with Cur-SMEDDS were evaluated for physical characteristics, emulsion droplet size, and curcumin content.

## 3. Results and Discussion

### 3.1. Alginate-Based Sponges Containing Curcumin-Loaded Self-Microemulsifying Drug Delivery Systems

**Figure 1:**
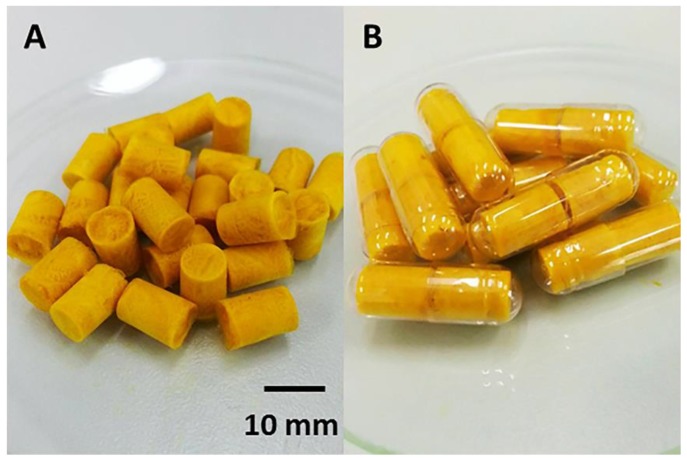
Macroscopic appearance of alginate-based sponges loaded with curcumin-loaded self-microemulsifying drug delivery systems (Cur-SMEDDS)—4% alginate, 2% hydroxypropyl methylcellulose (HPMC) A15 LV and 15% Cur-SMEDDS. (**A**) The prepared sponges; (**B**) Sponge inserts in gelatin capsules.

The highly porous alginate-based sponges containing curcumin-loaded SMEDDS were yellow in color (Figure 1) and highly flexible.

#### 3.1.1. Alginate Sponges

Alginate-based sponges were prepared using a 3%–5% *w*/*w* alginate solution with variable amounts of Cur-SMEDDS to enable selection of the best formulation, by evaluation of physical appearance and drug loading capacity. The sponges prepared using 3% *w*/*w* alginate solution displayed oil leakage when the Cur-SMEDDS loading exceeded 10% *w*/*w* (Table 1). The use of a higher concentration alginate solution (4% and 5%) was effective in preventing oil leakage and in enhancing the loading of liquid Cur-SMEDDS to a level of 20% *w*/*w*.

As expected, the sponge weight increased as the concentration of alginate solution and Cur-SMEDDS loading in the formulation increased (Table 1). The weight of the majority of sponge preparations was associated with values of standard deviation below 2, which confirmed the high degree of process uniformity.

The curcumin loading of alginate-based sponges increased as expected, with increasing amounts of curcumin-loaded SMEDDS in the formulation (Table 1), reaching a maximum of 15% *w*/*w* when the sponges were prepared using a 4% *w*/*w* alginate solution. High curcumin loading efficiencies between 80% and 92% were achieved. Sponges comprising 4% alginate were considered the optimum formulation in the present study, since the resultant sponges exhibited no oil leakage, uniform weight, high curcumin loading (15% *w*/*w*), and the maximum observed mean entrapment efficiency of 92%.

#### 3.1.2. Alginate-Colloidal Silicon Dioxide Sponges

Alginate-colloidal silicon dioxide sponges were composed of a 4% *w*/*w* alginate solution and variable amount of Cur-SMEDDS and colloidal silicon dioxide. The optimal ratio of Cur-SMEDDS and colloidal silicon dioxide that yielded the maximum Cur-SMEDDS loading in sponges was determined. The weight variation, curcumin content, and entrapment efficiency of the alginate-based sponges incorporating colloidal silicon dioxide are presented in Table 2. Colloidal silicon dioxide has been utilized previously as an adsorbent for S-SMEDDS. Sermkaew et al. [12] developed *Andrographis paniculata* SMEDDS pellets which contained up to 35% of the liquid form, and Setthacheewakul et al. [11] prepared Cur-SMEDDS pellets that could load liquid SMEDDS up to 37%. In the present study, the maximum Cur-SMEDDS loading of 25% in alginate-colloidal silicon dioxide sponges was achieved by the sponge formulation comprising 4% alginate and Cur-SMEDDS to colloidal silicon dioxide at the ratio of 10:1. The use of colloidal silicon dioxide decreased curcumin loading or loading efficiency compared with alginate-based sponges (Table 1). Therefore, colloidal silicon dioxide was excluded from the formulations. In this case, the high amount of adsorbent could completely adsorb the SMEDDS; nevertheless, the alginate to colloidal silicon dioxide ratio declined, resulting in the inability to transform the mixture to a sponge. In addition, Bertram et al. [34] defined that lyophilization did not change the physical state of their polymers, and larger amounts of the crystalline polymer as colloidal silicon dioxide resulted in an inability to form sponges after freeze-drying. In a preliminary study of alginate-based sponges on drug release, curcumin was released by more than 50% in first hour. Thus, additional polymers would be a good choice to modify release of sponges in further study.

#### 3.1.3. Alginate-Based Composite Sponges

Alginate-based composite sponges loaded with Cur-SMEDDS (15% *w*/*w*) were produced using a 4% *w*/*w* alginate solution and variable amounts of additional polymer (SCMC, HPMC A15 LV, and HPMC A4C provided in solution) to enable selection of the optimal controlled release formulation, evaluated by drug release. Composite sponges were produced by combining (1) alginate and SCMC or (2) alginate and HPMC. Both HPMC A15 LV and A4C had a degree of methoxy substitution of 29.5% (by weight) and the average viscosity at 20 °C of each 2% (by weight) aqueous solution was 15 and 400 mPa·s respectively. Oil leakage was not observed in any of the formulations. Curcumin content declined with increasing polymer (Table 3). These results showed that drug could be substituted with the polymer since its addition resulted in lower drug content. An increase in the amount of polymer added increased the curcumin encapsulation efficiency to around 96% in composite sponges containing SCMC or HPMC A15 LV.

### 3.2. Morphology and Pore Size of Composite Sponges

**Figure 2:**
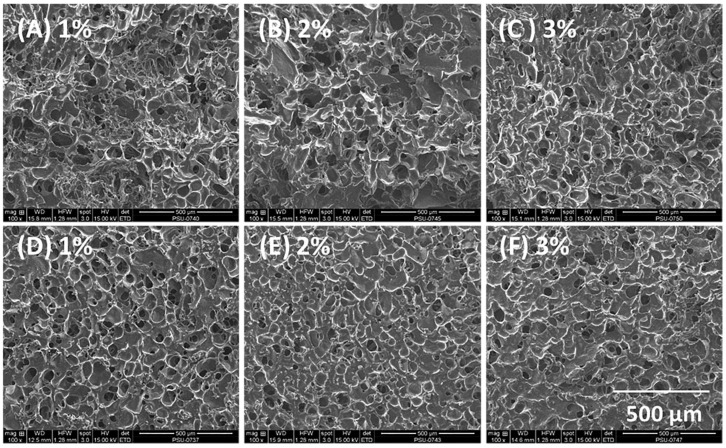
Scanning electron micrographs of composite sponges produced using 4% alginate and 1%–3% HPMC A15 LV with 15% curcumin-loaded SMEDDS. (**A**–**C**) Internal and (**D**–**F**) external morphology of sponges.

Scanning electron micrographs of composite sponges containing HPMC A15 LV revealed a highly porous surface and internal structure with roughly spherical pores of dimensions less than 100 µm, as shown in Figure 2. The average pore diameter was obtained from measurements of 100 pores, and measured 72.3 ± 7.2, 56.4 ± 6.8, and 40 ± 5.8 µm for composite sponges containing 1%, 2%, and 3% *w*/*w* HPMC. The reduction of pore size with increasing content of HPMC in the composite sponge simply correlated with the higher sponge density and is similar to the effect of increased carrageenan content on sponge structure reported by Bertram et al. [34].

### 3.3. Measurement of the Self-Microemulsifying Drug Delivery Systems Droplet Size in Composite Sponges

The average droplet size of curcumin microemulsions—which were extracted from lyophilized composite sponges containing 2% *w*/*w* HPMC by stirring in SGF at room temperature for 8 h—was 29.9 ± 0.2 nm. This value was comparable to the droplet size in liquid Cur-SMEDDS formulations. The small polydispersity index value of 0.089 ± 0.010 indicated the uniformity of the microemulsion droplets encapsulated within the sponge. The composite sponges provided resultant microemulsion droplet sizes of less than 50 nm. This demonstrated that the sponges are effective solid microemulsion delivery systems.

**Figure 3:**
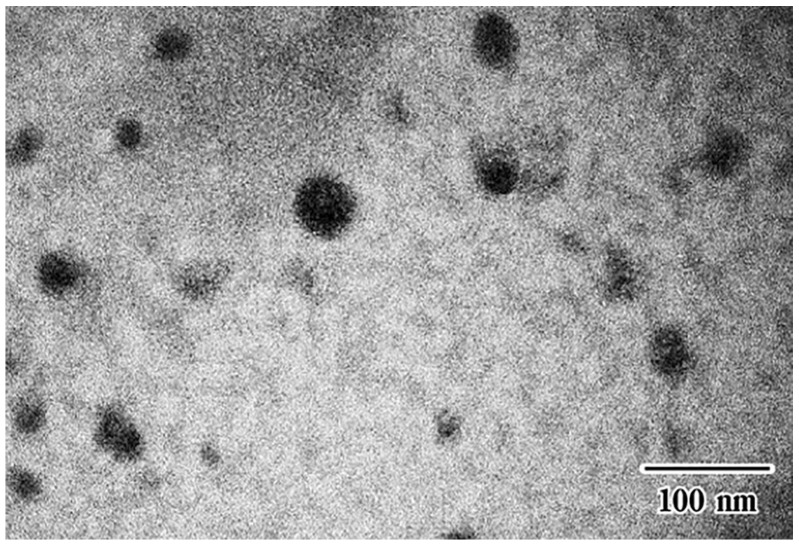
Transmission electron microscopy (TEM) micrograph of composite sponges.

Transmission electron microscopy micrographs of the Cur-SMEDDS microemulsion extracted from composite sponge containing 2% *w*/*w* HPMC A15 LV revealed that the droplets were almost spherical in shape (Figure 3). This finding confirmed that sponge preparation using lyophilization did not affect the emulsion droplet size, similar to other S-SMEDDS formulations as pellets [11], tablets [12], and other sponges [14].

### 3.4. Sorption Behavior of Composite Sponges in Simulated Gastric Fluid

**Figure 4:**
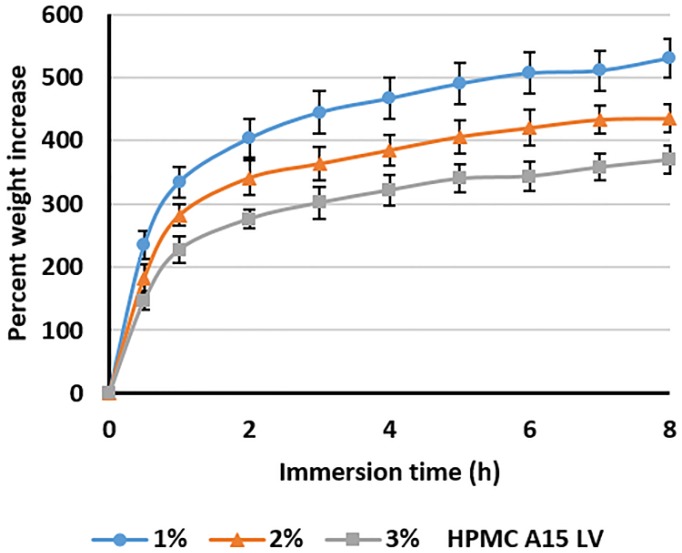
The percent weight increase of composite sponges containing 4% alginate, 1%–3% HPMC A15 LV, and 15% Cur-SMEDDS following immersion in simulated gastric fluid. Values represent the mean ± SD (*n* = 20).

The highly porous composite sponges—containing 4% alginate, 1%–3% HPMC A15 LV, and 15% Cur-SMEDDS—hydrated rapidly in SGF in less than 30 min, and uptake of SGF proceeded gradually over 8 h, resulting in sample weight increases of 350%–550% with decreasing HPMC content of the sponge, from 3% to 1% *w*/*w* (Figure 4). This behavior may be explained by the higher density of the polymer network within the sponge, which impedes fluid penetration.

### 3.5. Buoyancy of Alginate-Based Sponges

Buoyancy studies were carried out using a magnetic stirrer. All sponge formulations floated immediately in SGF at 37 °C and remained afloat for over 8 h. Thus, the buoyancy characteristics of the sponges were not influenced by the amount or type of polymer used in the preparation.

### 3.6. In Vitro Release of Curcumin from Alginate-Based Sponges

**Figure 5:**
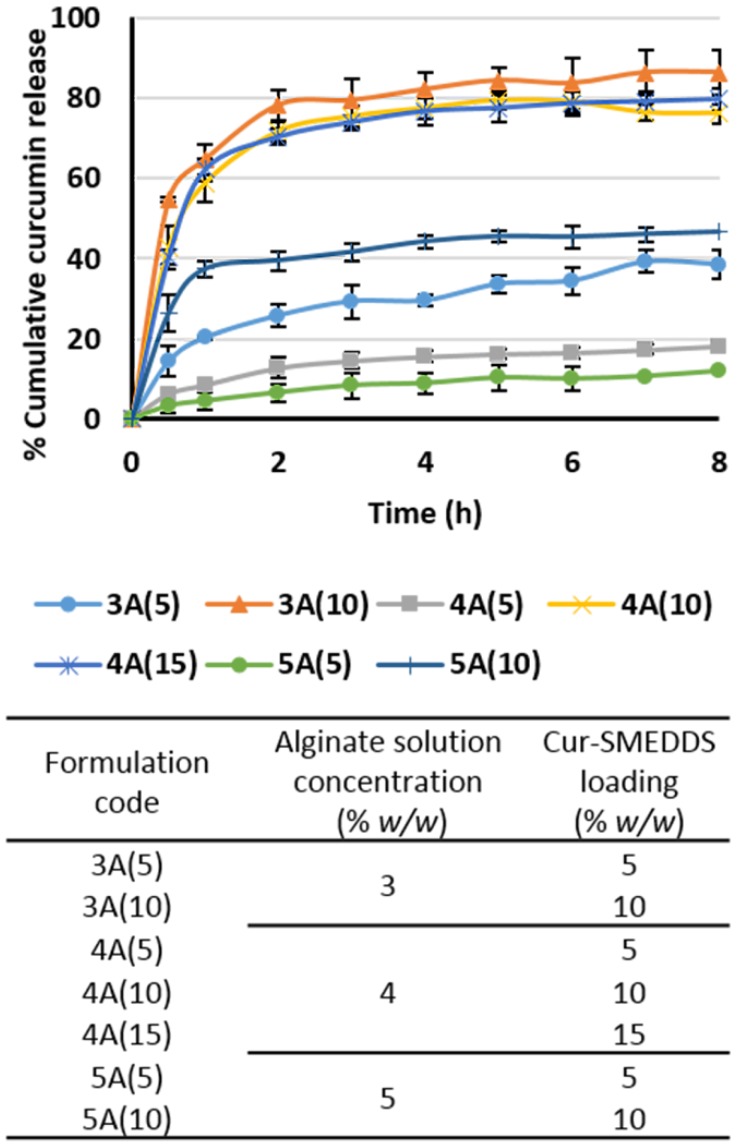
Release profiles of curcumin from alginate-based sponges in simulated gastric fluid at 37 °C. Data represent the mean ± SD (*n* = 3).

From Figure 5, cumulative curcumin release from alginate-based sponges containing 3%–5% alginate and 5%–10% Cur-SMEDDS—formulation 3A(5), 4A(5), 5A(5), and 5A(10)—were low and limited to 40%. Cumulative release of curcumin from alginate-based sponges using 3%–5% alginate with 10%–15% Cur-SMEDDS—formulation 3A(10), 4A(10), and 4A(15)—was much higher, approximately 80%. A possible explanation for this is that the lower curcumin content of these sponges provided low curcumin release. In a prior study [13,35], drug release was retarded when using a high-viscosity alginate and also with increased amount of alginate in the matrices. Moreover, permeation of the solution into the matrices may be obstructed because the higher polymer content provided a smaller pore diameter, as mentioned earlier.

Adjustments to the formulation were made based on the amount of liquid SMEDDS, the maximum drug release, and physical properties. Alginate-based sponges containing 3% alginate and 10% *w*/*w* Cur-SMEDDS achieved a maximum curcumin release of 90% in 8 h. However, the formulation was not considered for further studies since it contained only 10% *w*/*w* of liquid SMEDDS.

The release profile of alginate-based sponges containing 4% alginate and 15% Cur-SMEDDS displayed no significant difference from that observed for alginate-based sponges, which used 4% alginate with 10% Cur-SMEDDS (around 80% in 8 h), but they did provide a greater Cur-SMEDDS loading capacity (15% *w*/*w*). Hence, alginate-based sponges containing 4% alginate and 15% Cur-SMEDDS were selected for further studies.

**Figure 6:**
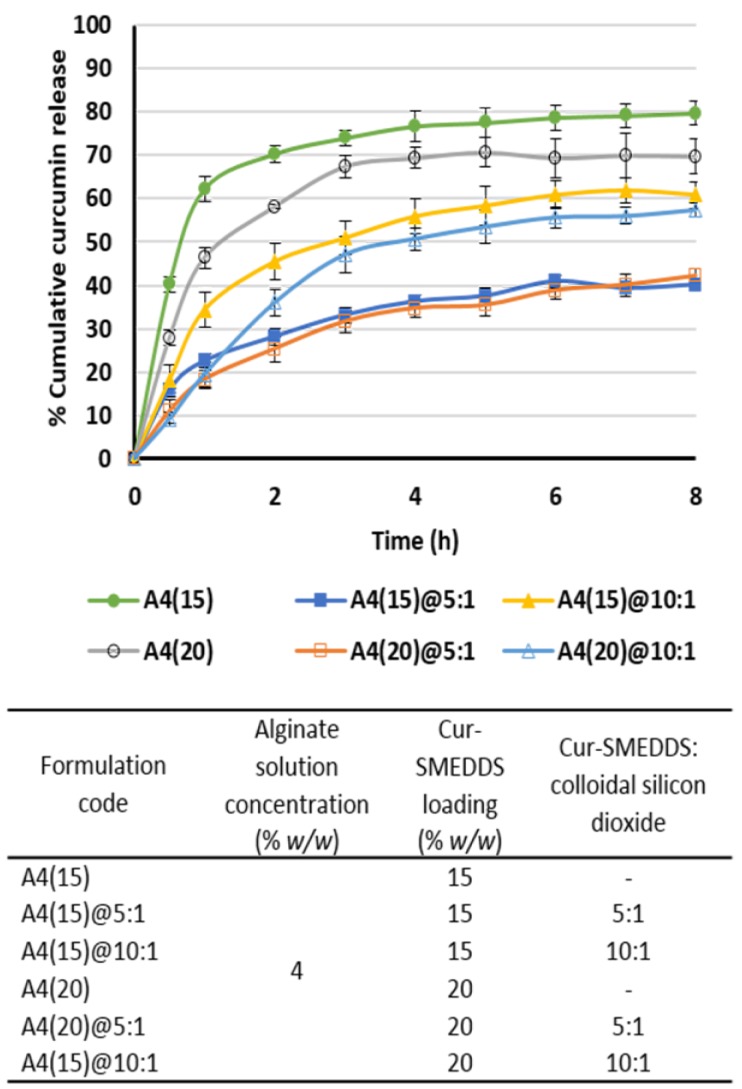
Effect of colloidal silicon dioxide addition on curcumin release from alginate-based sponges. Data are presented as the mean ± SD (*n* = 3).

Colloidal silicon dioxide was used in this study as an adsorbent to increase the SMEDDS loading capacity of the sponges. Colloidal silicon dioxide was able to increase the drug loading capacity because of its high specific surface area (200 m^2^/g) [36]. However, the presence of colloidal silicon dioxide at a SMEDDS:adsorbant ratio of 5:1 and 10:1 had little effect on the release of curcumin at 15% and 20% *w*/*w* curcumin loading (Figure 6). Cumulative release was limited to around 40% and 70%, respectively, in 8 h. However, it is of note that alginate-based sponges without colloidal silicon dioxide resulted in a higher cumulative curcumin release of 70%–80% in 8 h, indicating that strong binding between curcumin and the adsorbent reduces drug delivery efficiency. This finding is consistent with the findings of Sadeghi et al. [37], who reported that the addition of 1% colloidal silicon dioxide to a physical mixture of propranolol and Eudragit RS resulted in a reduction in drug release to 60% into distilled water over 20 min (particle size 250–300 µm).

**Figure 7:**
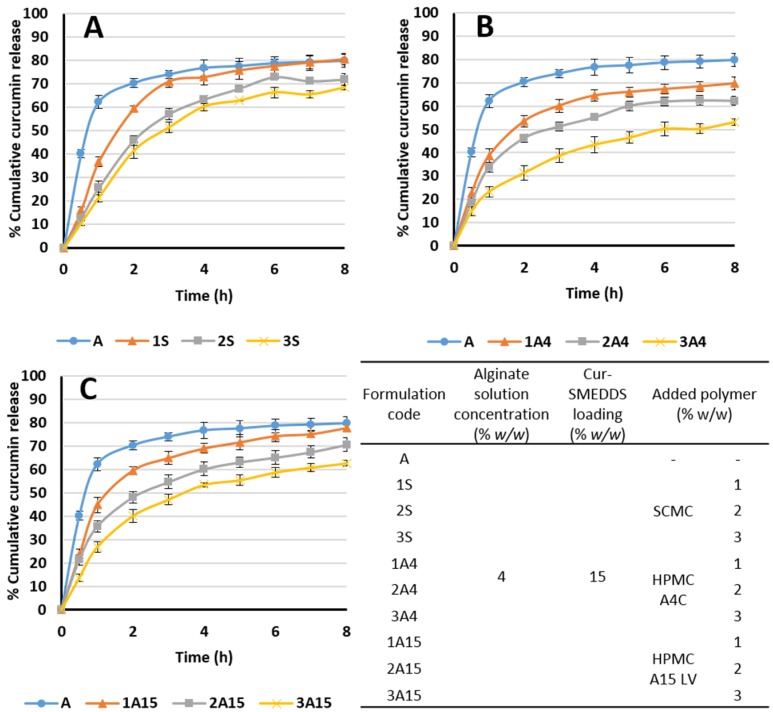
Effect of added polymer (A: sodium carboxymethyl cellulose (SCMC), B: HPMC A4C, and C: HPMC A15 LV) on the release of curcumin from alginate-based composite sponges. Data are presented as the mean ± SD (n = 3).

Sodium carboxymethyl cellulose (SCMC), HPMC A15 LV, and HPMC A4C respectively were included in alginate-based sponges loaded with Cur-SMEDDS to investigate the effect on curcumin release profiles. All three added polymers reduced the release of curcumin from the composite sponges, with increased amounts of the polymer resulting in decreased curcumin release. Composite sponges using 4% alginate and 3% SCMC exhibited cumulative curcumin release of 70% in 8 h compared with approximately 50% from composite sponges containing 4% alginate and 3% HPMC A4C (Figure 7). The viscosity of HPMC A4C is higher than HPMC A15 LV (400 and 15 mPa·s, respectively) resulting in slower drug release from alginate–HPMC A4C than from alginate–HPMC A15 LV composite sponges. Thus, curcumin release from alginate-based sponges loaded with Cur-SMEDDS may be controlled by varying the type and amount of SCMC and HPMC polymers. In common with several other controlled drug delivery systems, which function due to polymer swelling and gelling behavior, drug transport through the composite sponge matrix will be retarded by higher polymer concentrations and higher polymer viscosities [12,13,35].

### 3.7. The Optimized Composite Sponges

**Figure 8:**
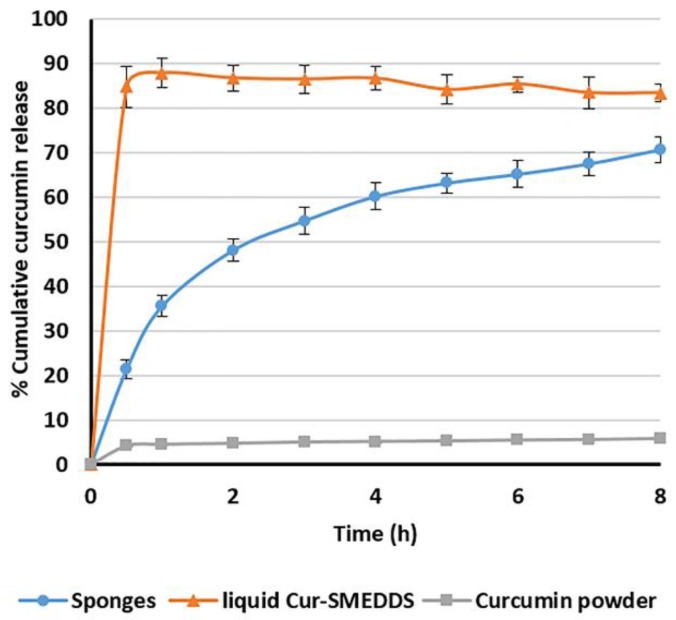
The release profile of curcumin from composite alginate/HPMC sponges loaded with Cur-SMEDDS, liquid Cur-SMEDDS, and curcumin powder in simulated gastric fluid at 37 °C. Data presented as the mean ± SD (n = 3).

The release profile in SGF of the optimized composite sponges loaded with Cur-SMEDDS (15% *w*/*w*) and prepared from 4% *w*/*w* alginate and 2% *w*/*w* HPMC A15 LV is compared with liquid Cur-SMEDDS and curcumin powder, as shown in Figure 8. Curcumin powder exhibited low aqueous solubility (30 pmol/mL) [7], resulting in less than 10% cumulative release in 8 h. As expected, liquid Cur-SMEDDS provided an immediate release (nearly 90%) in 1 h, since they rapidly formed an *o*/*w* microemulsion when in contact with the dissolution medium. Consequently, only novel developed alginate-based composite sponges loaded with Cur-SMEDDS showed sustained release properties, and reached 71% in 8 h. These results corroborate the ideas of Shishu et al. [32], who suggested that buoyant formulations (floating tablets of curcumin β-cyclodextrin (β-CD) complex) could sustain release of curcumin from the matrix tablets. Only 50% of curcumin was released in 5 h and almost completely released in 24 h. For the release pattern of plain curcumin and curcumin floating tablets, both release profiles were very low (2.25% in 24 h), whereas curcumin released from the curcumin β-CD complex was around 90% in 2 h.

In this case, the HPMC A15 LV gel formed when the sponges contacted water, and the gel performed as a barrier to the controlled release system. To explain the release of curcumin from the polymer-based matrix sponges, a fraction of curcumin could penetrate through the small holes while some also remained inside the matrices, since the alginate-based sponges are insoluble in acid solutions. It is possible that it might be completely released in gut lumen after alginate degradation.

An oral dose of curcumin at 20–80 mg/kg twice daily has been reported in the treatment of gastric ulcers in rats [38], whereas no exact dosage has been reported for humans. Although the developed sponge systems in this study contained a relatively low drug content of 3 mg/capsule, the amount of curcumin released was approximately 10 times greater compared to the plain curcumin, as shown in Figure 8. In addition, with the prolonged contact time of curcumin with the gastric ulcers for 8 h compared to the normal retention time of plain curcumin in the stomach (1–2 h), the sponge systems are expected to enhance the treatment efficacy. However, further research in animal models and clinical studies are needed to evaluate the potential use of this new delivery system in humans.

To examine the release kinetics of the optimized formulation, the release data obtained from in vitro drug release studies were plotted in various kinetic models: zero-order, first-order, and Higuchi’s model [39]. From the data analysis in Table 4, the Higuchi models achieved the highest correlations (*R*^2^ = 0.9546) with obtained data, which were plotted as cumulative percentage drug release versus square root of time. Higuchi kinetics equation describes dissolution of modified release pharmaceutical dosage forms. This indicated that the release of curcumin from the polymeric matrices seemed like a diffusion-controlled mechanism. The aqueous medium could infiltrate into microporous structure, leading to formation of *o*/*w* microemulsion at the surface and inside of sponges. Therefore, curcumin was mostly released by diffusion through the microporosity of sponges.

The stability of the “optimized” sponge formulation was evaluated in terms of physical appearance, emulsion droplet size, and curcumin content after 6 months of both intermediate (30 ± 2 °C/65% ± 5% RH) and accelerated storage conditions (45 ± 2 °C/75% ± 5% RH), and were found to be acceptable. No changes in physical appearance, including color and oil leakage, of the sponges were observed after stability study. Curcumin content decreased by less than 3% under both storage conditions after 6 months. The emulsion droplet size increased by only 8.7% (from 30 nm to 32.6 nm) after 6 months under accelerated storage conditions (Table 5), demonstrating the high physicochemical stability of the Cur-SMEDDS-loaded sponges.

## 4. Conclusions

Cur-SMEDDS were loaded in composite sponges based on alginate and HPMC using a freeze-drying method. The sponges floated in SGF for 8 h, and exhibited gradual and sustained release of around 70% of the curcumin content in 8 h, compared with less than 10% from curcumin powder, and immediate release of 90% from liquid Cur-SMEDDS formulations. These findings demonstrate the potential of composite sponges as gastroretentive delivery devices for SMEDDS formulations of curcumin and other poorly water-soluble compounds.

## Figures and Tables

**Table 1:** Weight, curcumin content, and entrapment efficiency of alginate-based sponges containing curcumin-loaded self-microemulsifying drug delivery systems (Cur-SMEDDS).

Alginate Solution Concentration (% *w*/*w*)	Cur-SMEDDS Loading (% *w*/*w*)	Oil Leakage	Weight (mg ± SD)	Curcumin Content (% *w*/*w*)	Encapsulation Efficiency (% ± SD)
3	5	no	19.1 ± 0.5	2.20 ± 0.02	83 ± 0.9
	10	no	30.6 ± 0.7	2.77 ± 0.03	85 ± 0.9
	15	yes	44.4 ± 1.1	3.21 ± 0.01	90 ± 0.3
	20	yes	56.1 ± 1.0	3.37 ± 0.01	91 ± 0.4
4	5	no	21.0 ± 0.3	1.88 ± 0.01	80 ± 0.5
	10	no	32.9 ± 0.3	2.66 ± 0.03	88 ± 1.0
	15	no	45.2 ± 0.8	3.09 ± 0.04	92 ± 1.2
	20	yes	58.5 ± 1.9	3.24 ± 0.03	91 ± 1.0
5	5	no	23.7 ± 0.6	1.68 ± 0.04	79 ± 1.8
	10	no	35.9 ± 0.6	2.46 ± 0.05	87 ± 1.9
	15	no	46.3 ± 3.5	2.85 ± 0.14	89 ± 4.4
	20	no	57.6 ± 5.5	3.07 ± 0.07	90 ± 1.9

SD: Standard deviation.

**Table 2:** Weight, drug content, and entrapment efficiency of the alginate-colloidal silicon dioxide sponges.

Alginate Solution Concentration (% *w*/*w*)	Cur-SMEDDS Loading (% *w*/*w*)	Cur-SMEDDS:Colloidal Silicon Dioxide	Oil Leakage	Weight (mg ± SD)	Curcumin Content (% *w*/*w*)	Encapsulation Efficiency (% ± SD)
4	15	5:1	no	54.6 ± 0.9	2.49 ± 0.01	86 ± 0.3
	20		no	69.4 ± 1.0	2.66 ± 0.01	87 ± 0.3
4	15	10:1	no	50.4 ± 0.8	2.68 ± 0.02	86 ± 0.8
	20		no	63.5 ± 0.7	2.90 ± 0.03	88 ± 0.8
	25		no	79.6 ± 0.7	2.96 ± 0.02	89 ± 0.6

**Table 3:** Weight, drug content, and entrapment efficiency of the composite sponges.

Alginate Solution Concentration (% *w*/*w*)	Cur-SMEDDS Loading (% *w*/*w*)	Added Polymer (% *w*/*w*)		Sponge Weight (mg ± SD)	Curcumin Content (% *w*/*w*)	Encapsulation Efficiency (% ± SD)
4	15	SCMC	1	48.4 ± 1.5	2.92 ± 0.03	88 ± 1.1
			2	51.3 ± 1.5	2.88 ± 0.02	89 ± 0.6
			3	53.3 ± 1.9	2.82 ± 0.03	97 ± 1.1
		HPMC A4C	1	47.1 ± 0.9	2.95 ± 0.05	85 ± 1.5
			2	51.7 ± 2.5	2.71 ± 0.02	87 ± 0.7
			3	54.0 ± 1.0	2.56 ± 0.01	88 ± 0.3
		HPMC A15 LV	1	47.6 ± 2.7	2.89 ± 0.05	91 ± 1.6
			2	49.5 ± 2.7	2.83 ± 0.04	93 ± 1.2
			3	52.5 ± 2.9	2.78 ± 0.02	96 ± 0.8

SCMC: sodium carboxymethyl cellulose; HPMC: hydroxypropyl methylcellulose.

**Table 4:** Correlation coefficient (*R^2^*) of the model equations applied to the release of the optimal formulation sponge (composite sponges loaded with Cur-SMEDDS (15% *w*/*w*), which were prepared from 4% *w*/*w* alginate and 2% *w*/*w* HPMC A15 LV) using various mathematical models.

**Kinetic Model**	Zero-order	First-order	Higuchi
	*Q_t_* = *K*_0_*t*	log*Q_t_* = log*Q*_0_ − *K*_1_*t*/2.303	*Q* = *K_H_* *t*^1/2^
*R***^2^**	0.7865	0.9009	0.9546

**Table 5:** Stability of alginate/HPMC composite sponges loaded with Cur-SMEDDS. Data are presented as the mean ± SD (*n* = 3).

Time (Months)	Oil Leakage	Emulsion Droplet Size (nm)	PDI	Curcumin Content (% *w*/*w*)
(A) 30 °C/65% RH				
0	no	29.9 ± 0.2	0.089 ± 0.010	100.5 ± 1.4
3	no	31.5 ± 2.0	0.125 ± 0.035	99.7 ± 2.3
6	no	31.2 ± 1.4	0.118 ± 0.050	97.4 ± 2.5
(B) 45 °C/75% RH				
0	no	29.92 ± 0.24	0.089 ± 0.010	100.5 ± 1.4
3	no	31.39 ± 1.44	0.139 ± 0.045	99.7 ± 2.0
6	no	32.60 ± 0.77	0.136 ± 0.070	98.1 ± 1.8

PDI: Polydispersity index; RH: relative humidity.
